# DNA methylation of RNA-binding protein for multiple splicing 2 functions as diagnosis biomarker in gastric cancer pathogenesis and its potential clinical significance

**DOI:** 10.1080/21655979.2022.2032965

**Published:** 2022-02-09

**Authors:** Ming Cheng, Xiaoan Zhan, Yi Xu, Saishan Wang, Hongcheng Zhang, Limin Fang, Hao Jin, Wei Chen

**Affiliations:** a Department of Gastroenterology, Zhejiang Jinhua Guangfu Tumor Hospital, Jinhua, Zhejiang, China; b Department of Gastrointestinal Surgery, Zhejiang Jinhua Guangfu Tumor Hospital, Jinhua, Zhejiang, China; c Department of Cardiology, Jinhua Fifth Hospital, Jinhua, Zhejiang, China

**Keywords:** Gastric cancer (GC), DNA methylation, RBPMS2, diagnostic significance

## Abstract

Higher methylation levels of RNA-binding protein for multiple splicing 2 (RBPMS2) was reported to be related with unfavorable outcome in gastric cancer (GC). However, molecular function and diagnostic significance of DNA methylation of RBPMS2 remains indistinct. Here we aimed to whether DNA methylation of RBPMS2 acts as a diagnosis biomarker in GC pathogenesis and its potential clinical significance. Western blot and immunochemistry assays were carried out to explore the level of RBPMS2. GC malignancy behaviors were determined by cell counting kit-8, Transwell, flow cytometry analysis and terminal-deoxynucleoitidyl transferase mediated nick end labeling staining. The inflammatory cell infiltration in xenograft model was observed by hematoxylin and eosin staining. CpG Islands was predicted by MethPrimer and the DNA methylation of RBPMS2 was evaluated by methylation-specific polymerase chain reaction. The results showed that RBPMS2 was downregulated in GC specimens. Poor survival rates were associated with low RBPMS2 expression. Overexpression of RBPMS2 inhibited GC growth while facilitated apoptosis in GC cells. In addition, level of DNA methylation of RBPMS2 in GC tissues was increased and DNA methylation of RBPMS2 was strongly associated with tumor invasion, Borrmann classification and TNM stage. We also observed that DNA methylation inhibitors counteracted the role of RBPMS2 in restraining GC development and tumorigenesis. To sum, our data demonstrated that DNA methylation of RBPMS2 was responsible for its downregulation in GC and promoted tumor progression, indicating DNA methylation of RBPMS2 might serve as a valuable potential parameter in GC pathogenesis.

## Graphical Abstract

**Figure:**
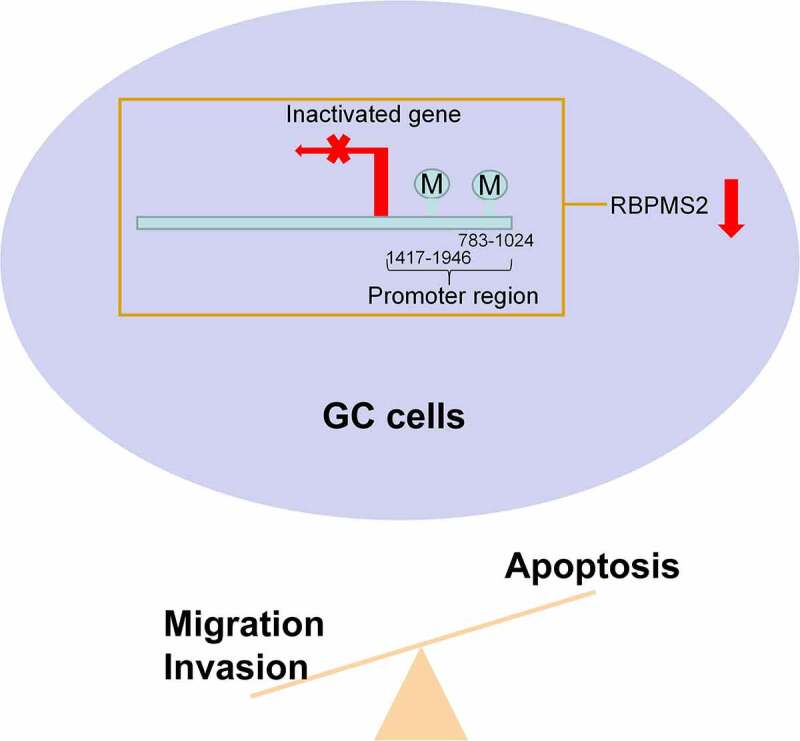

## Introduction

Gastric cancer (GC) is one of the malignancies with the highest incidence and mortality. The effectiveness and outcome of GC are strongly associated with the stage at which patients were diagnosed [1]. Therefore, early detection and therapy of GC are of the utmost importance to improve the quality of life and outcome of patients. Currently, the commonly used clinical screening methods for GC mainly include two problems: (1) The operation is more complicated and the compliance is relatively low, such as gastroscopy; (2) The detection accuracy rate is relatively low using biomarkers such as serum carcinoembryonic antigen, CA19-9, gastrin 17 and so on [2,3]. Hence, a more convenient and effective method for the early detection of GC was urgently needed. Additionally, initiation of GC is closely related to many factors including environmental stimuli, infection and genetical factors [4,5]. Therefore, in-depth exploration of abnormally expressed molecules and their regulatory mechanisms during the onset and development of GC and the active search for new therapeutic targets are of great importance to improve the therapeutic effect and prognosis of GC patients.

DNA methylation means that under the action of DNA-methyl transferase (DNMT), S-adenosylmethionine is used to provide a methyl donor, and its methyl group is transferred to the deoxycytosine ring. The 5-position carbon atom forms a covalent modification of methylated deoxycytosine [6–8]. DNA methylation can achieve a relatively stable and heritable apparent modification of DNA, and can regulate gene transcription, thereby causing gene silencing [9,10]. Accumulating studies have shown that DNA methylation is linked to embryonic development and the appearance and progression of tumors and diseases [10]. DNA methylation also plays an essential role in the onset and development of GC, and DNA methylation detection has broad prospects in the early diagnosis, prognosis, treatment and other clinical applications of gastric cancer [11]. The low methylation of CDKN2A gene, cg03079681, cg04026675, cg07562918, and cg13601799 locus predicts a better prognosis in GC [12]. Lian indicated that specific DNA methylation sites may well present the heterogeneity of colorectal cancer tissues, conducive to tailor treatment and accurate prediction of outcome [13]. Xin *et al*. indicated that silencing of miR-7-5p by DNA-methylation promoted GC stem cell metastasis via facilitating Smo and Hes1 expression [14]. However, whether the specific genes that occurred DNA methylation could act as a candidate for GC diagnosis still remains obscure.

Peng *et al*. depicted that higher expression of methylation of Sodium Channel Epithelial 1 Beta Subunit (SCNN1B), nuclear factor, erythroid 2-like 3 (NFE2L3) and Claudin 2 (CLDN2), RNA-binding protein for multiple splicing 2 (RBPMS2) was associated with unfavorable prognosis of GC patients [15]. Of them, the expression of RBPMS2 was significantly downregulated and with shorter survival of GC after 75 months. However, the function and diagnostic significance of RBPMS2 DNA methylation in GC remains indistinct. Therefore, this study intends to explore the function and diagnostic significance of RBPMS2 DNA methylation in GC.

## Materials and methods

### Cell culture and specimen collection

Human GC cell lines MGC-803, AGS, SNU-1, HGC-27, KATO III, and the normal gastric epithelial GES-1 cell line were purchased from the ATCC (USA) and kept in RPMI-1640 medium supplemented with 10% fetal bovine serum (FBS, Gibco, Carlsbad, CA, USA) and 1% penicillin-streptomycin at 37°C in an atmosphere moistened to 5% CO_2_. A total of 80 human tissues of GC and matched non-tumor adjacent tissues were gathered from patients who were pathologically diagnosed and underwent surgical section at Zhejiang Jinhua Guangfu Tumor Hospital between January 2019 and May 2019. Sample size was determined by power analysis using the G.power software. All patients provided informed consents, and all experiments were conducted following the Helsinki Declaration with the approval of the Ethics Committee of Zhejiang Jinhua Guangfu Tumor Hospital.

### Vectors transfection

The full length and the short harpin RNA (shRNA) of RBPMS2 provided by GenePharma (Shanghai, China) were cloned into pcDNA3.1 vector to construct Over-RBPMS2 or Sh-RBPMS2 plasmids. The plasmids were then transfected into MGC-803 or KATO III cells via Lipofectamine 2000 reagent (Invitrogen) according to the brochure provided by the producer.

### Quantitative real-time polymerase chain reaction (qRT-PCR)

Total RNA was extracted from cells and tumors using TRIzol reagent (Invitrogen). cDNA was synthesized from 20 μg RNA by reverse transcription using PrimeScript RT (Takara) reagent. The qRT-PCR was carried out on the CFX96 real-time PCR detection system (Bio-Rad, Hercules, CA, USA) using the SYBR Premix Ex Taq II (Takara) kit. The relative expression of target genes was detected by the 2^−ΔΔCt^ method [16]. Primers for qRT-PCR were listed as following: RBPMS2 (Forward): 5′-CTCCCATGCTGCGTTCA-3′, RBPMS2 (Reverse): 5′-GGGTGGTGTCAGA GGAAG-3′; glyceraldehyde phosphate dehydrogenase (GAPDH) (Forward): 5′-TCATTTCCT GGTATGACAACGA-3′, GAPDH (Reverse): 5′-GTCTTACTCCTTGGAGGCC-3′; U6 (Forward): 5′-AATACAGAGAAAGTTAGCACGG-3′, U6 (Reverse): 5′-GAATGCTTCAAAGAGTTGTGC -3′.

### Western blotting

Cellular or tissue proteins were extracted using a RIPA buffer containing 10% protease inhibitor (Roche) and quantified using a BCA kit. The proteins were then separated using a 10% sodium dodecyl sulfate polyacrylamide gel electrophoresis and transferred to a polyvinylidene fluoride membrane. The membranes were then probed with the primary antibodies, anti-RBPMS2 (1:1000, Abcam) and anti-GAPDH (1:2000, Cell Signaling Technology) at 4°C overnight. Finally, the membrane was incubated with secondary antibodies conjugated to horseradish peroxidase at 37°C for 60 min. Bands from immunoreactive protein bands were detected using electrochemiluminescence reagents and quantified using the ImageJ software. GAPDH acted as the internal reference.

### Cell counting kit-8 (CCK-8) assay

Cell proliferation was conducted using a CCK-8 reagent kit (Dojindo, Kumamoto, Japan) as per the manufacturer’s protocols. Briefly, MGC-803 and KATO III cells that underwent different transfections for 12, 24, and 48 hours were harvested, and 10 μL of CCK-8 reagent was added and cultured for another 2 hours. Next, optical density (OD) values were detected at 450 nm to analyze cellular proliferation capacity.

### Transwell assay

Transwell Chamber system (Corning) pre-coated with or without 1 mg/ml of Matrigel (Corning, NY, USA) was utilized to reflect GC invasive or migratory abilities, respectively. In short, MGC-803 and KATO III cells were treated with basal media for 24 h and added to the upper chamber with serum-free medium. Eight hundred μL of medium containing 30% FBS were introduced into the lower Chamber and the medium was removed from the upper Chamber. Non-migratory or noninvasive cells were wiped with a cotton swab after incubation in an incubator at 37°C with 5% CO_2_. The remaining cells were fixed, stained and washed with 4% paraformaldehyde, 1% purple crystal solution and phosphate buffer saline (PBS), respectively. The images were photographed and counted under an optical microscope.

### Flow cytometry analysis

Cell apoptosis and cell cycle distribution were detected by flow cytometry. In short, cells were seeded in 6-well plates and cultured for 48 h. Next, the cells were harvested, trypsinated, washed and resuspended in 200 μL of binding buffer. The cells were then treated with 5 μL of AnnexinV-fluorescein isothiocyanate (FITC) and 5 μL of propidium iodide (PI) in the dark for apoptosis detection. Cells were resuspended in PI master mix (40 mg/ml PI and 100 mg/ml RNase in PBS) at a density of 5 × 10^5^ cells/mL and incubated at 37°C for 30 min. Finally, cell apoptosis or cell cycle distribution was measured using a FACSCalibur (BD) flow cytometer.

### DNA methylation analysis

The CpG Islands were predicted by MethPrimer online tool (http://www.urogene.org/cgi-bin/methprimer/meth-primer.cgi). The bisulfite sequencing method was used to examine the methylation status of the RBPMS2 promoter region. In short, the genomic DNA was extracted using a DNA isolation kit and modified with bisulfite with the EZ DNA methylation kit (Zymo Research), and then amplified by PCR. PCR products were cloned in the T Easy vector and some positive clones were randomly selected for sequencing. Primer sequences were as listed: CpG, methylation-specific (forward): 5’-TATTAGTCCTTCGA GTAGTTATGAC-3’, and CpG, methylation-specific (reverse): 5’-AAATCGACCAACCAT CACTCACG-3’; unmeth ylation-specific (forward): 5’-CCATAGTTTTTTGAGTAGTACCG-3’, and unmethylation-specific (reverse): 5’-CATTACAAATAATTCACTTC-3’.

### Methylated DNA immunoprecipitation

Total DNA was extracted with a tissue DNA isolation kit (Omega Bio-Tek, Norcross, GA, USA) according to the manufacturer’s instructions. The concentration, integrity and purity of DNA sample was determined by NanoDrop 2000 c Spectrophotometer. Three microgram of DNA (3 μg) was sonicated into fragments (200–500 bp) using a sonicator (Sonics, Newtown, CT, USA). Fragmented DNA (1 μg) was denatured to produce single-stranded DNA. Immunoprecipitation was performed overnight at 4°C using 2 μg of anti-5mC antibody (ab10805, Abcam) or nonspecific human IgG antibody (ab6715, Abcam) as a negative control. DNA-antibody complexes were conjugated with protein A/G beads (Santa Cruze). After cross-link reversal and proteinase K treatment, immunoprecipitated DNA was extracted with phenol-chloroform, ethanol precipitated, treated with RNAse and purified. The harvested DNA fragments were resuspended in 10 μL of Tris buffer, and 1 μL of the DNA was used for RT-PCR detection.

### In vivo tumor growth model

Male BALB/c mice (aged 6 weeks, weight 18 ~ 22 g) were obtained from Vital River Co. Ltd (Beijing, China) and fed in a specific pathogen-free environment. The mice were randomly divided into 4 groups, including Control, 5-aza-DC, Sh-RBPMS2 and Sh-RBPMS2 + 5-aza-DC group. 1 × 10^6^ MGC-803 cells were subcutaneously injected into the mice. Tumor size was examined every 7 days with a caliper (volume = shortest diameter [2] × longest diameter/2). After 28 days, the mice were sacrificed, and the tumors were kept at −80°C until further analysis. This study was conducted in accordance with the National Institutes of Health’s Guide for the Care and Use of Laboratory Animals and was approved by the Ethics Committee for Animal Experimentation at Zhejiang Jinhua Guangfu Tumor Hospital. The pathological changes were detected by hematoxylin and eosin (H&E) staining assay.

### Immunohistochemistry assay

Mice tissues were fixed in formalin, embedded in paraffin and cut into 5 μm slices. The slices were then incubated with anti-Ki-67 at 4°C overnight after being washed with PBS and incubated with endogenous peroxidase blockers for 10 min. Next, the sections were incubated with anti-mouse IgG antibody labeled with horseradish peroxidase for 20 min at 37°C. The sections were incubated with 3,3’-diaminobenzidine and subjected to microscopic examination using OLYMPUS BX43 (200×) (PerkinElmer).

### In vivo apoptosis analysis by terminal-deoxynucleoitidyl transferase mediated nick end labeling (TUNEL) staining

TUNEL assay was utilized to evaluate apoptosis *in vivo* according to the manufacturer’s protocols. 4’,6-diamidino-2-phenylindole (DAPI) was used to stain cell nuclei. The images were obtained and photographed with an Olympus IX51 fluorescence microscope.

### Statistical analysis

The statistical analysis was carried out using SPSS 21.0 software and data were presented as the mean ± standard deviation. Comparisons between two or among more groups were detected via Student’s t-test or one-way analysis of variance (ANOVA) followed by Tukey’s post-hoc test. The receiver operating characteristic (ROC) curve was established to evaluate the diagnostic value of RBPMS2. A P value of less than 0.05 was considered to be statistically significant.

## Results

### RBPMS2 was decreased in GC tissues and its low expression predicted showed poor survival in GC patients

**Figure 1.**
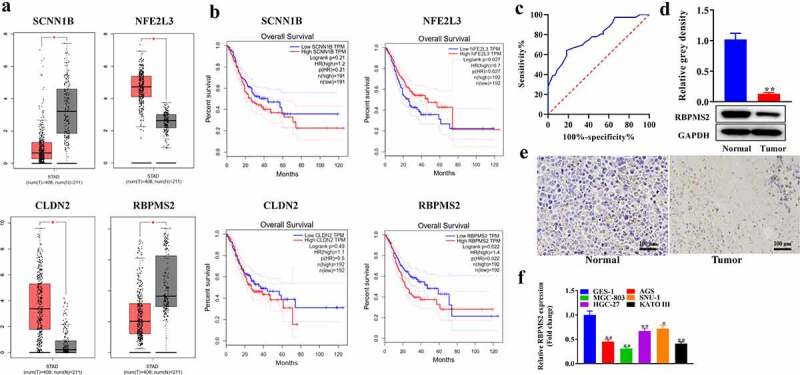
**RBPMS2 was downregulated in GC tissues and *its low expression predicted* poor survival in GC patients**. (a) The expression of SCNN1B, NFE2L3, CLDN2 and RBPMS2 in 408 GC tissues and 211 normal samples was obtained from the GEPIA website, which matches TCGA and GTEx data; *, P < 0.05 vs Normal group; (b) Associations of SCNN1B, NFE2L3, CLDN2 and RBPMS2 expression with the overall survival of GC patients were obtained from the GEPIA website; (c) The sensitivity and specificity of RBPMS2 for GC prediction was evaluated through ROC curve analysis. (d) Western blot assay was conducted to detect the expression of RBPMS2 in the GC tissues, **, P < 0.01, vs Normal group. (e) IHC was performed detect the expression of RBPMS2 in GC tissues; (f) qRT-PCR analysis of RBPMS2 in GC cell lines and control GES-1 cell line. *, P < 0.05, **, P < 0.01, vs GES-1 group.

Peng *et al*. revealed that higher methylation levels of four genes were associated with shorter survival of GC patients, including SCNN1B, NFE2L3, CLDN2, and RBPMS2. We explored the expression of these genes in GC and the relationship between the expression of these genes and the survival rate of GC patients using the GEPIA website (http://gepia.cancer-pku.cn/). The results displayed that the level of SCNN1B and RBPMS2 was significantly reduced while the expression of NFE2L3 and CLDN2 was increased in GC tumor tissues compared to paracarcinoma tissues (Figure 1a). Further survival curves were obtained from the GEPIA database. Median group cutoff was used. Ninety-five percent confidence interval (CI) is used as dotted line. Hazard ratio (HR) was calculated based on the Cox PH model). Data presented that GC patients with low NFE2L3 expression had a lower survival rate, while GC patients with low RBPMS2 expression had shorter survival after 75 months (Figure 1b). The ROC curve indicates that RBPMS2 can be used as a diagnostic biomarker for GC. The sensitivity is 65.89%, specificity is 81.82% and the area under the ROC is 0.7778 with the 95% CI of 0.6764–0.8791 (p <0.0001) (Figure 1c). Therefore, RBPMS2 was selected for further study. The Western blot and IHC tests showed that RBPMS2 expression was prominently reduced in GC tissues compared to non-tumor paracarcinoma specimens (Figure 1d,e). RBPMS2 was also downregulated in GC cell lines MGC-803, AGS, SNU-1, HGC-27, KATO III than control GES-1 cell line (figure 1f).

### Overexpression of RBPMS2 inhibited GC cell proliferation, invasion, and migration while promoted apoptosis

**Figure 2.**
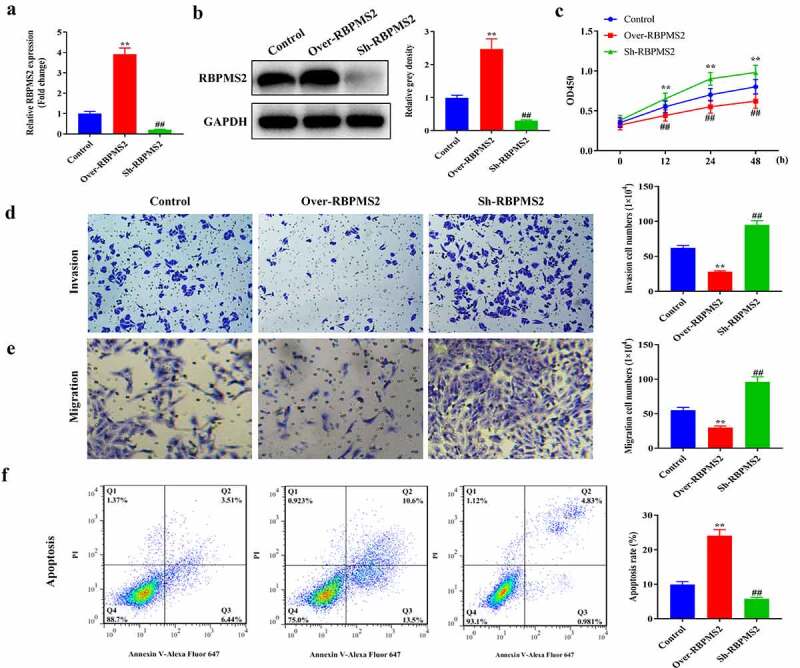
**Overexpression of RBPMS2 inhibited GC cell growth *in vitro***. (a and b) The expression of RBPMS2 in the MGC-803 cells was evaluated by qRT-PCR and Western blot; (c) CCK-8 assay was utilized to determine the MGC-803 cell proliferation affected by RBPMS2; (d and e) Transwell assay was conducted to examine the migration and invasion capabilities of MGC-803 cell affected by RBPMS2; (f) Flow cytometry assay was performed to determine the apoptosis rates affected by RBPMS2. **, P < 0.01, Over-RBPMS2 group vs NC group; ##, P < 0.01, Sh-RBPMS2 group vs NC group.

To understand the role of RBPMS2 in the progression of GC, the pcDNA3.1 plasmids of RBPMS2 overexpression and RBPMS2 silencing were transfected into MGC-803 cells. As illustrated in Figure 2a,b, RBPMS2 expression was significantly elevated or downregulated in MGC-803 cells compared to the NC group. The CCK-8 results indicated that upregulation of RBPMS2 inhibited the proliferative ability of MGC-803 cells while the suppression of RBPMS2 significantly promoted the proliferation of MGC-803 cells (Figure 2c). The Transwell assays indicated that migration and invasion of MGC-803 cells were significantly inhibited when RBPMS2 was overexpressed, while they were promoted when RBPMS2 expression was suppressed in MGC-803 cells (Figure 2d,e). On contrast, the apoptosis rates were greatly promoted in Over-RBPMS2 group while inhibited in sh-RBPMS2 group compared with NC group (figure 2f).

### The level of DNA methylation of RBPMS2 in GC tissues was increased

**Figure 3.**
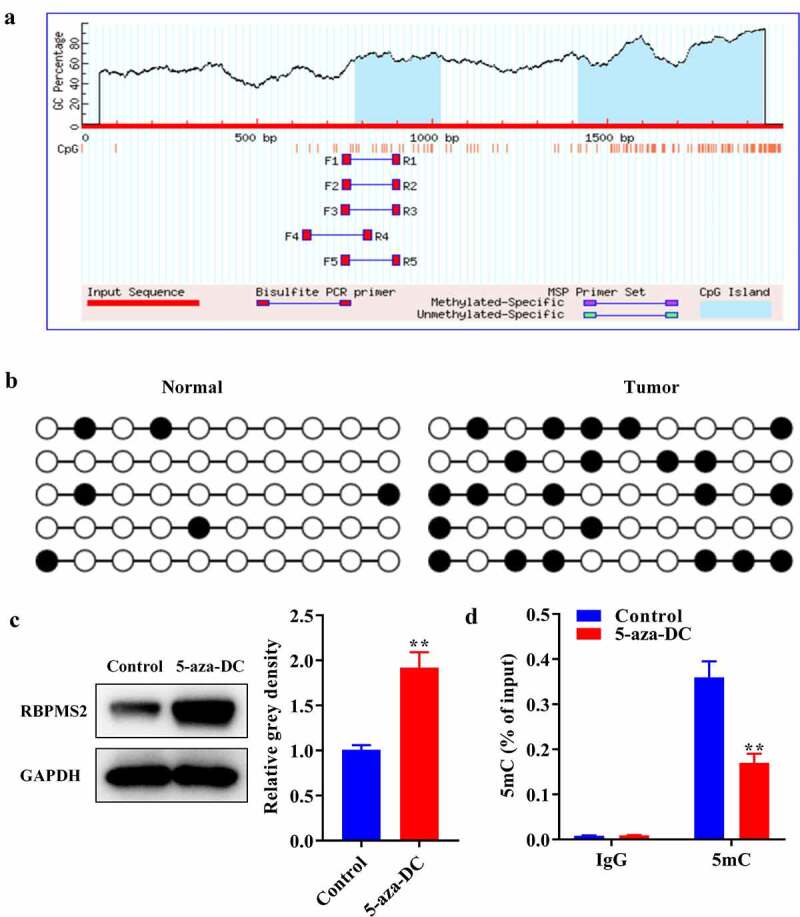
**The level of DNA methylation of RBPMS2 in GC tissues was increased**. (a) MethPrimer was used to detect the CpG Island in the promoter of RBPMS2; (b) MSP was utilized to detect the DNA methylation level of RBPMS2 promoter; (c) Western blot was performed to examine the expression of RBPMS2 in MGC-803 cells treatment with DNA methylation inhibitors 5-aza-DC; (d) Methylated DNA immunoprecipitation assay was performed to reveal the enrichment of 5mC in RBPMS2 promoter. **, P < 0.01, vs Control group.

To find out if the decrease in RBPMS2 expression was due to DNA methylation in the RBPMS2 promoter, MethPrimer was used to detect the CpG islet in the RBPMS2 promoter. The result showed that there are two CpG Islands in the promoter of RBPMS2 of 783–1024 and 1417–1946 (Figure 3a). The Island located at 1417–1946 which showed higher GC contents was used for DNA methylation measurement by MSP. MSP results indicated that the DNA methylation expression of RBPMS2 promoter was significantly enhanced in GC tissues in relation to non-tumor specimens (Figure 3b). In addition, we found that RBPMS2 expression was significantly increased in MGC-803 cells after treatment with 5-aza-DC, a DNA methylation inhibitor (Figure 3c). The 5mC level of the RBPMS2 promoter was significantly decreased in MGC-803 cells after 5-aza-DC pretreatment (Figure 3d). These results demonstrated that the downregulation of RBPMS2 was caused by DNA methylation at the epigenetic level. In addition, we found that RBPMS2 DNA methylation was related to the depth of tumor invasion, Borrmann classification, and TNM stage (Table 1). The results of multivariate COX regression analysis suggested that RBPMS2 can be used as an independent risk factor for the prognosis of GC patients (Table 2). These results demonstrated that DNA methylation of RBPMS2 was an important diagnostic marker in GC.

**Table 1.** DNA methylation of RBPMS2 in patients

Variable	Patients (n = 80)	RBPMS2 methylation			P value
		U (%)	P(%)	M (%)	
Age (years)					
< 60	24	8(33.3)	5(20.9)	11(45.8)	0.277
> 60	56	14(25.0)	13(23.2)	29(51.8)	
Gender					
Male	45	11(27.5)	13(32.5)	16(40.0)	0.464
Female	35	9 (25.7)	11(31.4)	15(42.9)	
Borrniann classification					
1 + 2	10	3(30.0)	4(40.0)	3(30.0)	< 0.001*
3	61	18(29.5)	11(18)	32(52.5)	
4	9	3(33.3)	2(22.2)	4(44.4)	
Tumor differentiation					
Well/moderate	25	5(20.0)	5(20.0)	15(60.0)	0.319
Poor	55	18(32.7)	5(9.1)	32(58.2)	
Tumor location					
Upper	8	0(0.0)	2(25.0)	6(75.0)	0.546
Middle	30	10(33.3)	10(33.3)	10(33.3)	
Lower	42	15(35.7)	11(26.2)	16(38.1)	
TNM stage					
I–II	25	10(40.0)	7(28.0)	8(32.0)	0.009*
III	40	9(22.4)	7(17.5)	24(60.0)	
IV	15	3(20.0)	2(13.3)	10(66.7)	
Invasion depth					
T1+ T2	37	26(70.3)	7(18.9)	4(10.8)	0.000*
T3+ T4	43	4(9.3)	5(11.6)	34(79.1)	
Lymph node nietastasis					
No	21	5(23.8)	6(28.6)	10(47.6)	0.754
Yes	59	16(27.2)	10(16.9)	33(55.9)	

Note: * means P value < 0.05.

**Table 2.** Multivariate analysis of the relationship between RBPMS2 expression and overall survival in GC patients

Variable	Multivariate analysis		
	HR	95%CI	*P*
Age	0.9099	0.8742–0.9455	0.504
Gender	0.9251	0.7745–1.0754	0.428
Borrniann classification	0.8853	0.7759–0.9947	0.739
Tumor differentiation	0.9049	0.7952–1.0145	0.417
Tumor location	0.7836	0.8127–1.0544	0.253
TNM stage	0.9895	0.9314–1.0475	0.258
Invasion depth	0.9206	0.8494–0.9918	0.319
Lymph node nietastasis	0.9931	0.9219–1.0643	0.504
RBPMS2	0.8334	0.7485–0.9184	<0.001*

Note: * means P value < 0.05.

### DNA methylation inhibitors reversed the function of suppressing RBPMS2 in GC cell malignant behaviors

**Figure 4.**
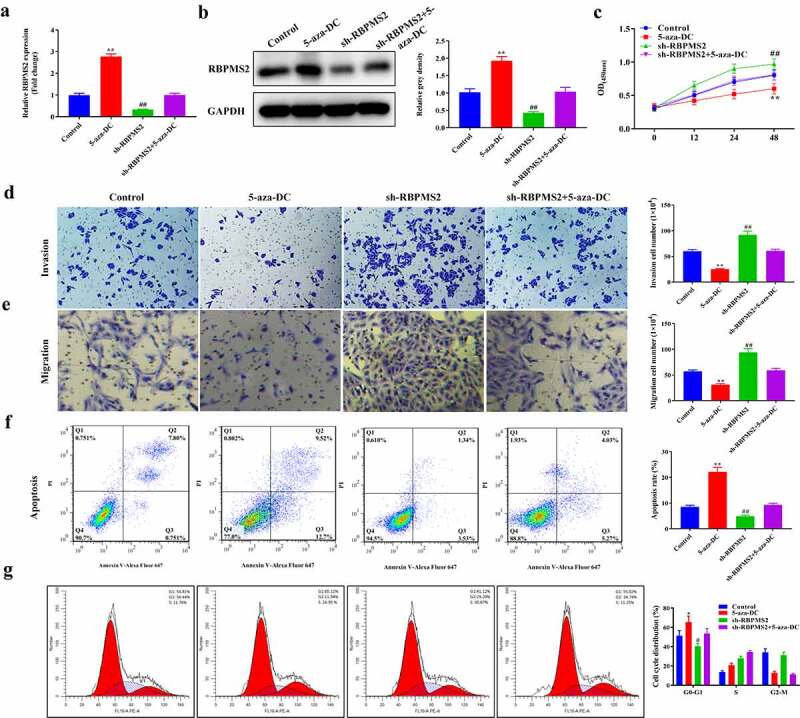
**5-aza-DC reversed the function of suppressing RBPMS2 in MGC-803 cell malignant behaviors**. (a and b) The expression of RBPMS2 in MGC-803 cells affected by 5-aza-DC was measured by qRT-PCR and Western blot; (c) CCK-8 was performed to evaluate the proliferation of MGC-803 cells affected by 5-aza-DC; (d and e) Transwell was conducted to determine the migration and invasion in RBPMS2-silenced MGC-803cells affected by the 5-aza-DC; (f) The apoptosis was evaluated by flow cytometry in RBPMS2-silenced MGC-803 cells affected by the 5-aza-DC; G, Effects of 5-aza-DC and sh-RBPMS2 on cell cycle distribution in MGC-803 cells were evaluated by flow cytometry. *P < 0.05, **P < 0.01, 5-aza-DC group vs NC group; #, P < 0.05, ##, P < 0.01, Sh-RBPMS2 + 5-aza-DC group vs Sh-RBPMS2 group.

**Figure 5.**
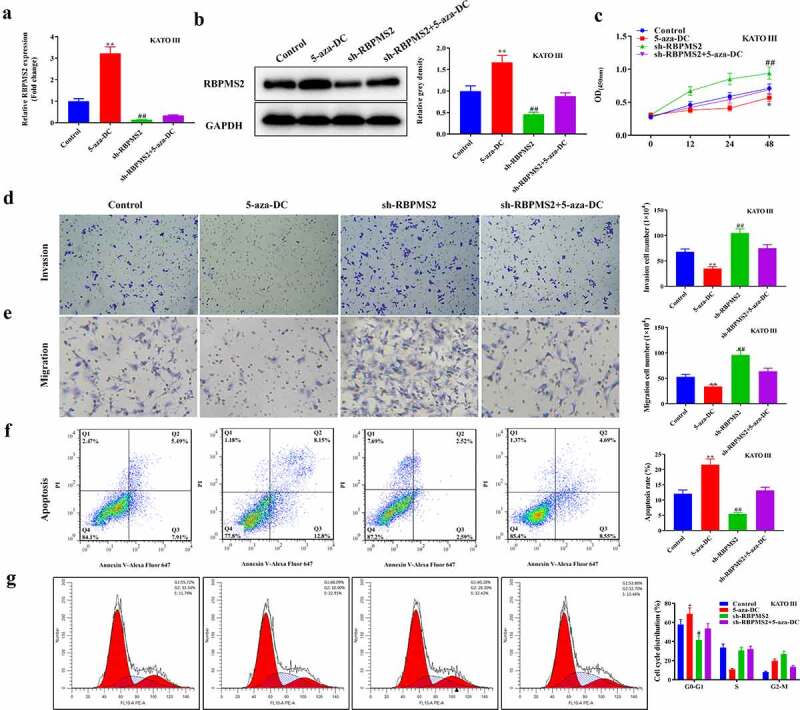
**5-aza-DC reversed the function of suppressing RBPMS2 in KATO III cell malignant behaviors**. (a and b) The expression of RBPMS2 in KATO III cells affected by 5-aza-DC was measured by qRT-PCR and Western blot; (c) CCK-8 was performed to evaluate the proliferation of KATO III cell affected by 5-aza-DC; (d and e) Transwell was conducted to determine the migration and invasion in RBPMS2-silenced KATO III cells affected by the 5-aza-DC; (f) The apoptosis was evaluated by flow cytometry in RBPMS2-silenced KATO III cells affected by the 5-aza-DC; (g) Cell cycle distribution of KATO III cells after treatment of 5-aza-DC and Sh-RBPMS2. *, P < 0.05, **, P < 0.01, 5-aza-DC group vs NC group; #, P < 0.05, ##, P < 0.01, Sh-RBPMS2 + 5-aza-DC group vs Sh-RBPMS2 group.

**Figure 6.**
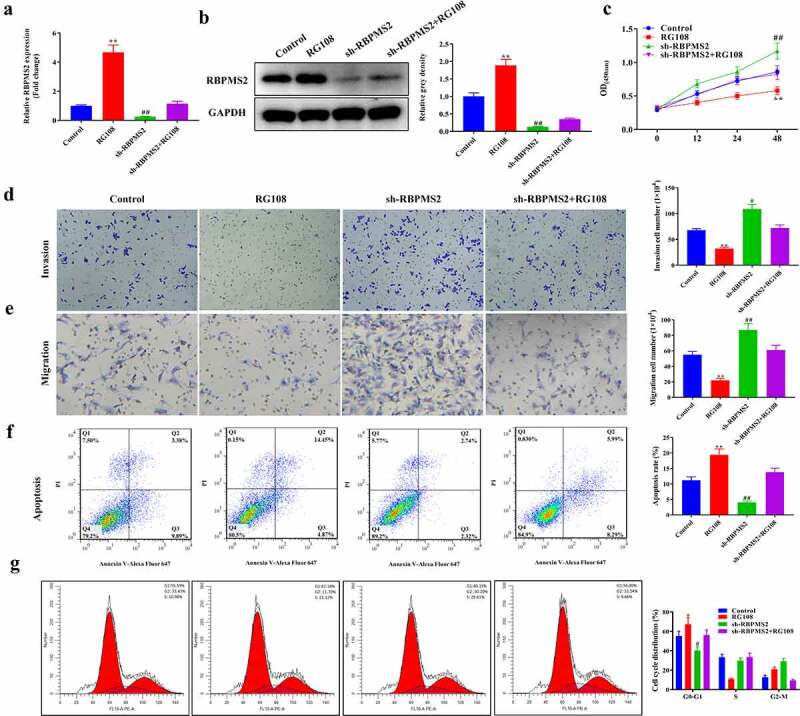
**RG108 reversed the function of suppressing RBPMS2 in MGC-803 cell malignant behaviors**. (a and b) The expression of RBPMS2 in MGC-803 cells affected by RG108 was measured by qRT-PCR and Western blot; (c) CCK-8 was performed to evaluate the proliferation of MGC-803 cells affected by RG108; (d and e) Transwell was conducted to determine the migration and invasion in RBPMS2-silenced MGC-803 cells affected by the RG108; (f) The apoptosis was evaluated by flow cytometry in RBPMS2-silenced MGC-803 cells affected by the RG108; (g) Cell cycle distribution of MGC-803 cells after treatment of RG108 and Sh-RBPMS2. *, P < 0.05, **, P < 0.01, RG108 group vs NC group; #, P < 0.05, ##, P < 0.01, Sh-RBPMS2+ RG108 group vs Sh-RBPMS2 group.

To better understand the role of DNA methylation in GC progression, a rescue assay was performed in RBPMS2-silenced MGC-803 cells by treatment with 5-aza-DC (Figure 4). As depicted in Figures 4a,b, RBPMS2 expression was significantly increased in the 5-aza-DC group compared to the control group. Meanwhile, the sh-RBPMS2 induced decrease in RBPMS2 expression was partially rescued by cotreatment with 5-zaz-DC. The CCK-8 and Transwell tests showed that proliferation, migration, and invasion were significantly inhibited in RBPMS2 cells by 5-aza-DC, while apoptosis rates were increased by 5-aza-DC group compared to the control group. Further analysis indicated that 5-aza-DC may also partially reverse the sh-RBPMS2-induced increase of the proliferation, migration, and invasion and the decrease of apoptosis in GC cells (Figure 4c,f). 5-aza-DC caused cell cycle arrest in G0/G1 phase and rescued the effects of sh-RBPMS2 on MGC-803 cell cycle distribution (Figure 4g). Another GC cell line KATO III was used and the same rescue effects of 5-aza-DC on sh-RBPMS2 were demonstrated (Figure 5). To further confirm the importance of RBPMS2 methylation in GC, another DNA methylation inhibitor RG108 was used. RG108 exerted the same effects as 5-aza-DC to reverse the function of suppressing RBPMS2 in GC cellular malignant behaviors (Figure 6). These results demonstrated that DNA methylation inhibitors reversed the function of suppressing RBPMS2 in GC cell proliferation, invasion, migration, apoptosis, and cell cycle arrest.

### DNA methylation inhibitors partially reversed the function of suppressing RBPMS2 on tumor growth in vivo

**Figure 7.**
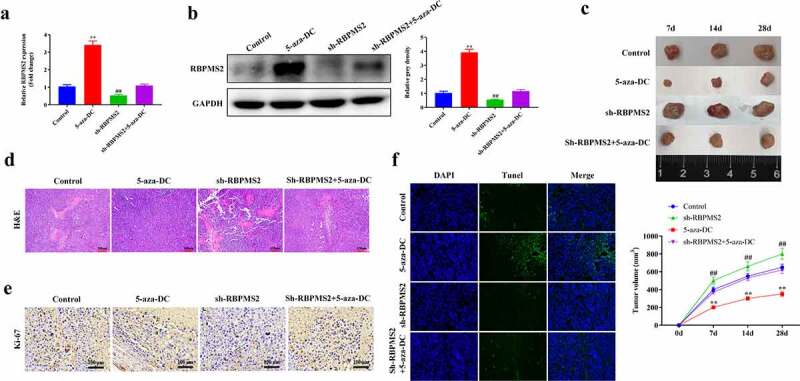
**5-aza-DC partially reversed the function of suppressing RBPMS2 on tumor growth *in vivo***. (a and b) The expression of RBPMS2 affected by 5-aza-DC in xenografts was evaluated by qRT-PCR and Western blot; (c) Tumor size affected by RBPMS2 and treatment with 5-aza-DC in sh-RBPMS2 group; (d) H&E assay was used to detect inflammatory cell infiltration in sh-RBPMS2 group and treatment with 5-aza-DC; (e) Ki-67 immunohistochemistry was performed to measure the proliferation of tumor cells affected by RBPMS2 and treatment with 5-aza-DC; (f) TUNEL staining was conducted to evaluate the apoptosis affected by sh-RBPMS2 group and 5-aza-DC; **, P < 0.01, Sh-RBPMS2 group vs NC group; ##, P < 0.01, Sh-RBPMS2 + 5-aza-DC group vs Sh-RBPMS2 group.

We also evaluated the role of RBPMS2 DNA methylation in GC tumor growth *in* *vivo* (Figure 7). RBPMS2 level was reduced in the sh-RBPMS2 group while the reduction of RBPMS2 could be partially restored by treatment with 5-aza-DC in sh-RBPMS2 group (Figure 7a,b). Besides, tumor size was evidently larger in the sh-RBPMS2 group in relation to the control group. However, the tumor size could be reduced by treatment with 5-aza-DC in sh-RBPMS2 group (Figure 7c). H&E assay showed that more serious inflammatory cell infiltration was observed in sh-RBPMS2 group while 5-aza-DC partially alleviated inflammatory cell infiltration in tumors (Figure 7d). In addition, Ki-67 immunohistochemistry and TUNEL staining test showed that proliferation was significantly promoted while apoptosis was overtly inhibited in the sh-RBPMS2 group compared with the control group (Figure 7e,f). On contrast, the function of RBPMS2 on proliferation and apoptosis of tumor cells could be counteracted in part by treatment with 5-aza-DC. Collectively, these data implied that DNA methylation inhibitor, 5-aza-DC, partially counterbalanced the function of suppressing RBPMS2 on tumor growth *in vivo*.

## Discussion

GC is one of the most common gastrointestinal neoplasms around the world, and its morbidity and mortality rank fourth and fifth among the world’s cancers, respectively [17]. China’s GC morbidity and mortality account for more than 50% in the world. At present, surgical resection is still the main method of GC treatment [18]. In China, more than 80% of patients are usually diagnosed at an advanced stage at first visit and the 5-year survival rate after surgery is less than 30%. Although much progress has been made in the treatment of GC in recent years, the survival rate of patients with GC remains very low [5]. Therefore, it is urgently needed to find new feasible indicators for early diagnosis of GC. In the present study, the results elucidated that DNA methylation of RBPMS2 was responsible for its downregulation in GC and promoted tumor progression, suggesting that DNA methylation of RBPMS2 was essential for the diagnosis of GC.

DNA methylation participated in gene transcription and post-transcriptional regulation, miRNA gene expression regulation and post-transcriptional regulation of long non-coding RNA and is closely related to tumorigenesis [11,19,20]. In fact, increasing numbers of studies also suggested that DNA methylation is implicated in regulating the progression of GC [6,10,21,22]. For example, Kim *et al*. reported that the CpG sites of HOXC10 are hypomethylated in GC samples and that upregulation of HOXC10 promoted cell growth in GC [23]. Bai *et al*. illustrated that DNA methylation-induced alteration of genetic signature is associated with overall survival rate in patients with GC, suggesting that these genes might be a prognostic nomogram for GC [24,25]. Zeng *et al*. estimated that DNA and histone methylations that alter gene expression in GC could be the new epigenetic therapy targeted in GC [26]. In the previous study, positive coefficients indicated that the higher methylation expressions of RBPMS2 was associated with shorter survival of GC patients, suggesting methylation of RBPMS2 as a biomarker for GC [15]. However, the function and the diagnostic value of GC remain unclear. In our study, MSP results indicated that RBPMS2 promoter region was extra methylated in GC tissues compared to the adjacent non-tumor specimens. RBPMS2 expression level was hindered by regional hypermethylation at promoter CpG Islands in GC tissues and showed close association with survival of GC patients. In addition, overexpression of RBPMS2 restrained GC cell growth and promoted apoptosis in GC cells. DNA methylation inhibitors 5-aza-DC and RG108 reversed the function of suppressing RBPMS2 in GC cell malignant behaviors, implying that DNA methylation of RBPMS2 leads to the downregulation of RBPMS2 in GC, and thereby promoted tumor progression. Methylation is catalyzed by DNMTs as a donor of methyl group [27]. ESRP1 is a RBPMS2-binding partner [28], Its expression is correlated with DNA methylation in ovarian cancer cells [29] and can regulate methylation in non-small cell lung cancer cells [30]. It was inferred that coordination of ESRP1/RBPMS2 interplay facilitates DNMT recruitment on DNA methylation.

In addition, several genes that are regulated by DNA methylation have been indicated as novel diagnostic biomarkers or new therapeutic targets [31]. For example, Amini *et al*. reported that significant CD40 hypermethylation was observed in breast cancer specimens in relation to non-tumor adjacent specimens, which was strongly associated with the clinical stage of malignancy, suggesting that CD40 DNA methylation in breast cancers is a novel epigenetic biomarker [32]. Chang *et al*. reported that NDRG2 methylation is related to the depth of tumor invasion, Borrmann classification, and TNM stage, suggesting that NDRG2 methylation may play an important role in breast cancer metastases [33]. In the current study, we also demonstrated that RBPMS2 DNA methylation was related to the depth of tumor invasion, Borrmann classification and TNM stage, suggesting that DNA methylation of RBPMS2 was a promising molecular diagnostic biomarker of GC. However, this should be further investigated in the future study.

## Conclusion

Taken all together, we demonstrated that RBPMS2 level was reduced in GC samples attributed to regional hypermethylation at promoter CpG Islands and that the survival rate of GC patients was low in case of low RBPMS2 expression. Further analysis showed that RBPMS2 DNA methylation was related to the depth of tumor invasion, Borrmann classification, and TNM stage. Overall, we demonstrated that RBPMS2 DNA methylation can inhibit tumor progression, suggesting that RBPMS2 DNA methylation functioned as a potential candidate for the diagnosis of GC.
